# Insights into *Leishmania* Molecules and Their Potential Contribution to the Virulence of the Parasite

**DOI:** 10.3390/vetsci8020033

**Published:** 2021-02-20

**Authors:** Ehab Kotb Elmahallawy, Abdulsalam A. M. Alkhaldi

**Affiliations:** 1 Department of Zoonoses, Faculty of Veterinary Medicine, Sohag University, Sohag 82524, Egypt; 2 Biology Department, College of Science, Jouf University, Sakaka, Aljouf 2014, Saudi Arabia

**Keywords:** *Leishmania*, parasite, virulence, factors

## Abstract

Neglected parasitic diseases affect millions of people worldwide, resulting in high morbidity and mortality. Among other parasitic diseases, leishmaniasis remains an important public health problem caused by the protozoa of the genus *Leishmania*, transmitted by the bite of the female sand fly. The disease has also been linked to tropical and subtropical regions, in addition to being an endemic disease in many areas around the world, including the Mediterranean basin and South America. Although recent years have witnessed marked advances in *Leishmania*-related research in various directions, many issues have yet to be elucidated. The intention of the present review is to give an overview of the major virulence factors contributing to the pathogenicity of the parasite. We aimed to provide a concise picture of the factors influencing the reaction of the parasite in its host that might help to develop novel chemotherapeutic and vaccine strategies.

## 1. Introduction

Leishmaniasis is a group of neglected tropical diseases caused by an opportunistic intracellular protozoan organism of the genus *Leishmania* that affects people, domestic animals and wild animals worldwide e [1,2,3]. Humans contract the infection mainly by the bite of female sand flies from the genera *Phlebotomus* in the Old World and *Lutzomyia* in the New World. The public concern about the disease is increasing due to the appearance of new endemic foci for the disease, potentiated by habit changes, climatic changes and the expanded range of sand fly vectors. This group of diseases is now found in 98 countries around the world, affecting a total of 12 million people [1,4,5], and approximately 350 million people are at risk for infection; in addition, an estimated 500,000–2,000,000 new cases and 20,000–50,000 deaths occur annually [3,4,5]. The disease has been linked to tropical and subtropical regions, in addition to being endemic in many areas worldwide [1,3].

**Figure 1:**
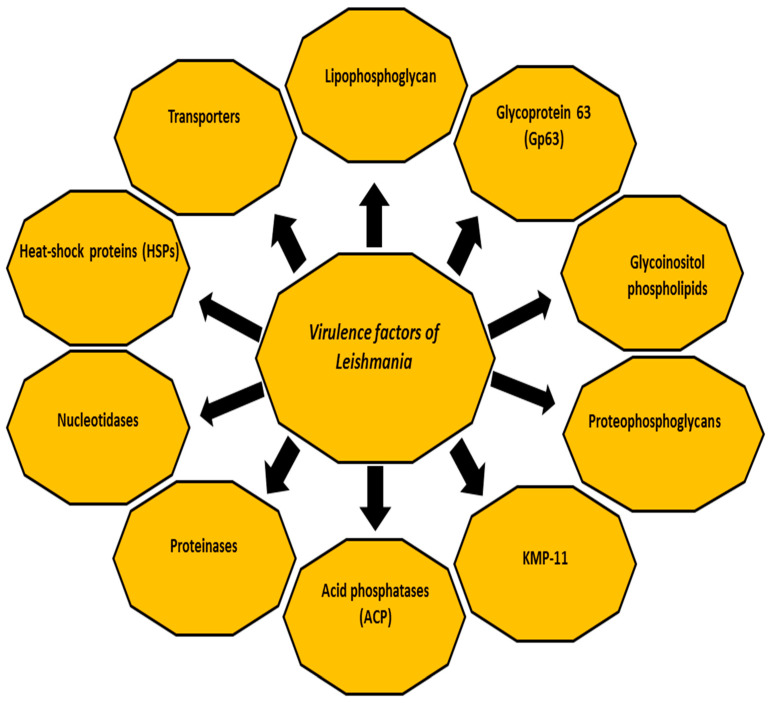
The major virulence factors of *Leishmania* species.

The three following forms of the disease are known, based on the infecting parasite species and host immune response: cutaneous leishmaniasis (CL), mucocutaneous leishmaniasis (MCL) and disseminated visceral leishmaniasis (VL) which has a fatal prognosis in the absence of treatment [6,7]. In accordance with the causative species, CL is caused by the following species: *Leishmania major (L. major)*, *L. tropica* and *L. aethiopica* in the Old World (Eastern Hemisphere, mainly Africa, Asia and Europe); whereas in the New World (Western Hemisphere, specifically the Americas) CL species are: *L. amazonensis*, *L. mexicana*, *L. braziliensis*, *L. panamensis*, *L. peruviana* and *L. guyanensis* [8,9,10,11]. In addition, CL is caused by *L. infantum* (synonymous of *L. chagasi*) in the Old World and New World. L MCL is caused by *L. braziliensis*, *L. panamensis* and *L. guyanensis* in the New World [8,9,10,11]. The final form (VL) is mainly caused by *L. donovani* complex (Africa, India and Asia), and *L. infantum* (synonymous of *L. chagasi*) in America, South America and the Mediterranean area [8,12], with 500,000 annual estimated cases [3]. Despite several studies on *Leishmania*, many questions remain unanswered. Among others, the control of the disease remains beyond our reach, particularly with increasing numbers of subclinical cases of *Leishmania* infections that may flare up due to immunosuppression or an existent source of infection in the context of blood transfusions or organ transplants. Early case detection followed by adequate treatment represents the key to controlling the disease, which may improve the prognosis and reduce transmission. Understanding the virulence factors of the infectious agent, the immunological mechanisms and the host immune response seems crucial for determining the course and the clinical outcome of any infection. Furthermore, better understanding the host immune response would be very helpful in seeking novel drugs, drug targets and vaccine preparation [13,14]. This review aims to summarise some facts about the virulence factors of *Leishmania* (Figure 1).

## 2. Virulence Factors of *Leishmania*

During its life cycle, *Leishmania* undergoes a series of morphological and biochemical changes in energy metabolism, protein degradation, motility and antioxidant and stress-related defences, making the parasite able to persist, replicate within macrophages and spread to establish the infection [15,16,17]. In fact, several studies have reported plenty of individual virulence factors contributing to the infectivity of *Leishmania* in their host and intracellular parasitism in addition to the inhibition of the host immune response. For example, both parasite and host proteinases affect the dynamics of the infection by *Leishmania* [18,19,20]. The classes of proteinases in *Leishmania* species include cysteine proteinases, metalloproteinases and serine proteinases [19,20]. The host proteinases, such as matrix metalloproteinases, play a crucial role in the subversion of the host immune response. Other virulence factors include glycoinositolphospholipids (GIPLs) [21,22,23], lipophosphoglycan (LPG) [24,25], proteophosphoglycan (PPG) [26], A2 protein [23,27], cysteine proteinases [23], surface acid proteinase (Gp63Gp63) [23,27] and 11 kDa kinetoplastid membrane protein (KMP-11) [28]. These factors might modulate the interaction between *Leishmania* and host immune cells [17,19,29]. The following sections briefly highlight the major virulence factors of *Leishmania* that contribute to the pathogenesis of the disease and enable the parasite to establish the infection [19,23,30,31,32].

### 2.1. Lipophosphoglycan

LPG is among the most abundant cell surface heterogenous glycoconjugate molecules; it is mainly present in the promastogote stage of the parasite and is strongly downregulated or absent on amastigotes [33]. LPG is a large molecule composed of two parts linked by a phosphodiester bond: an oligosaccharide backbone coated with repeating disaccharide phosphate units (Galb1, 4Man-PO4), attached to a glycan core and inserted into the membrane by an inositol anchor (glycosylphosphatidylinositol, GPI) which is mainly made up of lipids [33]. Although the two parts are conserved among *Leishmania* species, the precise structure of LPG varies depending on the species and stage of the parasite [33,34]. The heterogeneity of LPG among stage and species seems associated with the substituents groups branched on the linear phosphoglycan chain (PG) and oligosaccharide cap [35]. These structures involved the modification among various stages of the life cycle and parasite metacyclogenesis [36]. In accordance with stage-specific variations of LPG, metacyclic promastigotes involve a higher number of repeating units of PG domain, and therefore they are significantly longer than the procyclic promastigotes [37]. On the other hand, the stage-specific variation of the oligosaccharide cap occurs through the replacement of the galactoside residue by an arabinopyranoside residue. Importantly, the changes occur in PG domains of LPG are among the main characteristic virulence factors that involve species variation. To our knowledge, three types of *Leishmania* LPG have been reported based on the nature of the side chain in the PG domain and the substitution sites. In *L. donovani*, LPG is linear with no substitutions in the PG [36], while LPGs of *L. infantum*, *L. major*, *L. mexicana*, and *L. tropica* are glycosylated in the linear PG at position C3 of the galactose [38]. On the other hand, LPGs are mannosylated in *L. aethiopica* at the C2 position of the mannose. Given the above information, sugar residues of the PG domain influence the heterogeneity of LPG. It is noteworthy to mention that several previous studies documented the intraspecific variability of LPG among the same species of *Leishmania* but from different field isolates [39]. It seems that these stage-specific polymorphisms and inter/intra species variations contribute to the parasite survival and are involved in the selectivity and competence of sand fly vectors for their specific strains [40,41]. A previous study documented that the structural polymorphism of LPG from *L. infantum* and *L. braziliensis* trigger the stimulation of host cells via Toll-like receptors (TLRs) [35]. In addition, other previous studies revealed that the LPG of dermotropic strains caused by *L. infantum*, which are devoid of side chains, triggers the higher production of cytokines and nitric oxide (NO) levels in comparison with that of viscerotropic strains, whereas LPGs target the immunosuppression by interaction with macrophages [42,43,44]. On the other hand, LPGs play a pro-inflammatory role during the infection by *L. amazonensis* and *L. braziliensis*. It should be stressed that LPG is largely confined to promastigote stages, absent or downregulated in amastigotes LPG. Its expression is developmentally modified on metacyclic promastigotes and these modifications are critical to its function [45,46].

In accordance with its function, LPG has been implicated in *Leishmania* pathogenesis by triggering TLRs 1 and 2, which are well-known signalling receptors mediating the activation signals in the cells of the innate immune response in mammals [47,48,49]. Furthermore, the tissue tropism of different *Leishmania* species is related to the variations in their surface glycolipids [50]. The LPG of promastigotes plays several roles, including resistance to the complement system, inhibition of the oxidative burst response, induction of the inflammation response and prevention of natural killer T cells from recognising the macrophage infected with *Leishmania* [51]. LPG also impairs the nuclear translocation of nuclear factor kappa-light-chain-enhancer of activated B cells (NF-κB) in monocytes, resulting in a subsequent decrease in interleukin (IL) 12 production [52,53,54]. In *L. infantum*, LPGs are considered a TLR2/TLR4 agonists besides and they induce the production of prostaglandin E_2_ and heme-oxygenase-1 [44,55]. Likewise, LPG can influence the immune responses of the host by modulating dendritic cells which in turns lead to the inhibition of antigen presentation and the promotion of earlier IL-4 response [56]. Interestingly, several studies used the mutants, e.g., *L. major* mutants deficient in LPG (lpg1 (-)), and these studies showed that the LPG1 gene plays an important role in the survival of the parasite in the *Phlebotomus duboscqi* insect vectors but not required for the survival of *Leishmania* in *Phlebotomus argentipes* and *Phlebotomus perniciosus* [51]. Similarly, a previous study used phosphoglycan (PG)-deficient mutant lpg2 (-) and revealed the critical role of Lpg2 for the survival of *L. major* in these three sandfly species [51]. Given this information, it seems that the structural polymorphisms of LPGs are evolutionary driven and control the vectorial competence of various phlebotomine sand flies for different *Leishmania* spp [57]. Another recent study on *L. infantum* also revealed that the deletion of *LPG2* impaired the outcome of infection in neutrophils [58]. The other actions of LPG include its involvement in the phagocytosis of promastigotes, the inhibition of lymphoproliferative response and the activation of T suppressor cells, and the protection of *Leishmania* from intralysosomal microbicidal factor [59,60,61].

### 2.2. Glycoinositolphospholipids (GIPLs)

GIPLs, mainly free GPIs, are well characterised for members of the Trypanosamitidae family. In *Leishmania*, both the major surface glycoprotein Gp63 and the abundant LPG are attached to the lipid bilayer by inositol-containing glycolipids. GIPLs are the predominant class of glycolipids synthesised by all developmental stages of *Leishmania* [19]. These molecules play an important inhibitory role in *L. major* survival inside macrophages by inhibiting inducible nitric oxide synthase (iNOS) and protein kinase C [17,20,62]. A clear correlation has also been reported between GIPL-containing detergent-resistant membrane domains of *L. (Viannia) braziliensis* and the rate of macrophage infection by this species [29,63,64]. Furthermore, a previous study documented that the inter- and intraspecies polymorphisms in LPGs and GIPLs are not only important for the interaction with hosts in the old world species of *Leishmania* but also in the New World species, revealing their role as a major key elements for the survival of the parasite inside the host vector in addition to their roles in the modulation of the host immune response as a result of infection [35,65]. These functions of the polymorphisms of LPG and GIPLs combined with their association to pro-inflammatory profile were confirmed in *L. enriettii* [66].

### 2.3. Proteophosphoglycans (PPGs)

PPGs are highly glycosylated polypeptides cover the plasma membrane of the parasite, forming a sausage-shaped structure-enclosed amastigote with O-glycosylations similar to those found in the LPG and acid phosphatase [24,67,68,69]. PPGs exist as secretory and surface-bound forms in both promastigotes and amastigotes in *Leishmania* [70]. They belong to serine- and threonine-rich *Leishmania* proteins that are extensively modified by phosphodiester-linked phosphooligosaccharides and terminal mannooligosaccharides [71]. The function of membrane PPGs remains unclear; however, they contribute to the establishment of the parasitophorous vacuole [71,72,73,74], and the activation of the complement [71,72]. Interestingly, the N-terminal domain of PPG has the potential of a DNA vaccine against experimental VL caused by *L. donovani* because it elicits a Th1-type immunoprotective response, represented by a surge in IFN-γ, tumour necrosis factor (TNF) α and IL-12 levels, in addition to the extreme downregulation of transforming growth factor (TGF) β, IL-4 and IL-10. In the same study, a rise in the level of *Leishmania*-specific immunoglobulin G2 (IgG2) has been reported, which is an indicator for enhanced cell-mediated immunity [75]. Likewise, PPGs seem important for the parasite colonisation of the sandfly, transmission and mammalian infection [26]. Among others, the filamentous proteophosphoglycan (fPPG) were reported to accompany the parasite species during transmission [76]. It should be stressed that the secretion of this filamentous gel was accompanied by the differentiation of mammal-infective transmission stages, confirming that the behavioural manipulation of the infected vector by *Leishmania* might provide a selective advantage to the parasite [77]. Moreover, a previous study has confirmed the role played by PPGs in different species of sand fly that were addressed using LPG2-deficient mutants [78]. In this study, PPGs were considered key molecules which target the resistance of the parasite to midgut digestive enzymes through the prevention of the killing of lpg2(-) promastigotes. Interestingly, PPGs accelerated the wound healing in the host infected by *L. mexicana* by the activation of macrophages which is driven by the action of insulin-like growth factor 1-dependent signalling [79]. Furthermore, a previous study revealed that *L. major* PPGs were expressed by amastigotes of the parasite and bound to macrophages, which resulted in the inhibition of the production of TNF-α, which together with IFN-γ, stimulated the production of NO by macrophages, modulating the biology of the infected immune cells [70].

Importantly, the secretion of mucin-like gel called promastigote secretory gel (PSG), which is comprised largely of PPGs, represents one of the adaptation mechanisms of *Leishmania* species for their sand fly vectors. This secretory gel is mainly localised in the mouth parts and midgut of sand fly vectors. Interestingly, PSG accelerated the transmission of the parasite through the enhancement of the regurgitation of metacyclic promastigotes during blood meal as a result of blocking these localised regions (stomach valve, anterior mid-gut, and mouth) [80]. More importantly, PSG greatly influences the action of macrophages and neutrophils recruitment at the site of infection and this action is usually allied with saliva [81]. The presence of macrophages with PSG might favour the survival of the parasite in the hostile environment and the persistence of the infection, in addition to their synergistic action with saliva and sand fly bite [82,83]. It is noteworthy to mention that PSG reduced the efficiency of the elimination of the parasite by inflammatory macrophages through their influence on the catabolism of L-arginine to NO, which represents one of the most effective mechanisms of parasite killing [84]. This catabolism occurs though the action of inducible nitric oxide synthase (iNOS). In addition, the extracellular L-arginine might also influence the adaptive immune response by affecting T cells proliferation and T cells receptor signalling, in addition to their role in the production of cytokines [85]. However, other studies documented that PSG targeted the alternative activation of macrophages through the enhancement of the expression and increasing the activity of arginase-1, explaining the possible competition between PSG and iNOS for L-arginine [81]. Furthermore, a previous report documented the critical role of proteophosphoglycan-rich gel of PSG from L. tropica, L. major, from *Lutzomyia longipalpis* which exacerbates the cutaneous lesions in mice together with parasite growth, reinforcing the hypothesis which proposes that these molecules are very crucial and evolutionarily conserved structures of Leishmania [86]. Given the above information, PPGs and PSG play a major role in the protection of the parasites against the proteolytic damage, favouring *Leishmania* transmission and the progression of the infection [26,78].

### 2.4. 11 kDa Kinetoplastid Membrane Protein (KMP-11)

KMP-11 is an 11 kDa kinetoplastid membrane hydrophobic protein associated with LPG and which has shown immunoregulatory properties [87,88,89]. It was found in many kinetoplastid parasites, including *Leishmania,* in both stages, and its surface expression increases during metacyclogenesis [90]. This hydrophobic protein also induces the expression of IL-10 in cells from patients with CL and MCL; however, the mechanism underlying these effects is still unclear [91,92]. Some previous reports have suggested the involvement of KMP-11 with the following functions: parasite mobility, attachment to the surface of the host cell, the stimulation of T-cell proliferation and the regulation of the cytoskeleton through interaction with the subpellicular microtubules [88,93].

### 2.5. Acid Phosphatases (ACPs)

Acid phosphatases (ACPs) form a group of enzymes released at both stages of *Leishmania*, particularly the promastigote stage [94,95,96,97]. The cell surface has two forms of ACP, membrane-bound and secretory, which are antigenically distinct [95,96,97,98]. Large quantities of ACP seem to participate in the pathobiology of the disease by eliciting the humoral immune response of the host, adaptation of the parasite in acidic environments and acquisition of nutrients from host cells [59,99]. The membrane-bound ACP reduces the respiratory burst of neutrophils and inhibits the toxic oxidative metabolite production of neutrophils; therefore, it favours the survival of parasites inside the host cell [59,100,101,102]. It also dephosphorylates certain phospholipids and phosphoproteins [100,103]. This ectoenzyme seems to protect *Leishmania* by inhibiting the production of superoxide anions by neutrophils and macrophages that produce microbial free radicals [100,104]. Moreover, some studies have linked extracellular acid phosphatase activity with the degree of promastigote infectivity/virulence [105].

### 2.6. Proteinases

Proteinases are among the most important virulence factors, playing central roles in the interaction between parasite and host [20]. They hydrolyse peptide bonds and degrade proteins and peptides [20,106]. In addition, they are involved in the steps of parasite invasion and migration inside the host, immune evasion, pathogenesis and disease outcome [107]. Proteinases can be classified based on their catalytic domains as serine-, threonine-, aspartyl-, metallo- and cysteine proteinases [108,109]. These groups of enzymes, proteinases, are associated with various pathogenic processes and mediate immunopathology during infection [110,111,112]. It should be stressed that several studies targeting genomic analysis have reported that the proteinases’ genes are kept constant in various *Leishmania* species; however, there is a high diversity of proteinases in the parasite [20]. Importantly, the function of these critical enzymes varies according to the infecting species [20,106]. Among others, aspartyl-, metallo- and cysteine-proteinase are the most studied proteinases in *Leishmania* species [113]. Cysteine proteinase (CP) appears to be localised in the megasome, a modified lysosome-like organelle observed in the stationary phase, in New World CL and the extracts of *L. major* amastigotes [111,114,115]. These enzymes have been also involved in mechanisms of survival and growth of amastigotes inside macrophages [110], in addition to their intracellular degenerative action for proteins, favouring intracellular parasite survival [20]. Among others, the most studied CPs in *Leishmania* were CPA, CPB and CPC. They belong to the group clan CA, which is then divided into two families; family C that includes cathepsin B-like that involves CPC, and cathepsin L-like that comprises CPA and CPB enzymes; while family C2 includes calpain-like enzymes [116]. In accordance with their functions, a previous study on *L. infantum* revealed that CPA is associated with the infection of mammalian hosts cells in vitro [117]. Meanwhile, the activities of CPB on mammalian hosts were different according to the infecting species. In this regard, CPB triggered the Th2 profile during the infection by *L. mexicana* in BALB/c mice, in addition to their role in the induction of lesions, the production of IL-4 and IL-5, and the inhibition of IL-12 and NO production by cleaving the STAT-1 and AP-1 transcription factors. Meanwhile, CPB targeted the Th1 profile in C57BL/6 mice and C3HeB/FeJ was infected by *L. mexicana*. CPB enhanced the expression of its associated cytokines [116,118,119,120,121]. In *L. chagasi* and *L. major*, CPB was reported to induce the Th1 profile and regulate the production of IFN-γ [120,122]. CPB is also associated with the following functions in *L. (L.) amazonensis;* cleavage of MHC class II gene, the induction of Th1 or Th2-related cytokines; as well as the activation and stimulation of CD8^+^ T lymphocytes [123,124]. Taken into account, there are several types of CPB, named type I, Type II and type III. Interestingly, CPB encoded the genes from different species of *Leishmania* in addition to their long lasting protection against the infection [125]. It should be stressed that Type I mainly comprises C- terminal extension (CTE) domain in Kinetoplastidae and several CP genes have been identified in various species of *Leishmania* e.g., *L. major*, *L. pifanoi* and *L. amazonesis* [126,127,128]. It seems that parasites contain multiple, highly active cysteine peptidases with many stage-regulated proteinases that modulate the host immune response [59,129]. However, it should be kept in mind that single nucleotide polymorphisms (SNPs) might arise in many of these CP genes during life cycle differentiation. In addition, CP genes might vary based on the infecting species and the parasitic stage. On the other hand, CPC enhances the expression of TGF-β during *L. (L.) chagasi* infection [130], while it contributes to the resistance in *L. (L.) mexicana* to get killed by macrophages [131,132].

The expression of aspartyl-proteinases changes between morphological forms and seems to be related to the host responses to survive in distinct micro-environments [133]. Recently, it was reported that *L. (Viannia) braziliensis* promastigotes express serine proteinases that have distinct subcellular distributions and expression. Taken together, this might contribute to the maintenance of this parasite’s lifestyle at physiological pH, in the cytosol and on the external face of the parasitic membrane [134]. In stark contrast, the proteinases secreted from the host also affect the dynamics and progression of the infection, in addition to the development of the lesion [135]. For example, matrix metalloprotease-9 (MMP-9) interferes with the re-epithelisation of chronic wounds in humans, where TNF-α and pro-inflammatory chemokines in regulation with MMP-9 delay normal wound healing [136,137]. A recent study reported that small myristoylated protein-3 is a potential virulence factor in *L. amazonensis* [138]. Taken together, MMP-9 plays a crucial role in tissue destruction and the excessive degradation of the basal membrane, the migration of inflammatory cells to the site of infection and ulcer development; therefore, therapeutic modulation of MMP-9 may be a useful approach for improving disease outcomes.

Interestingly, several previous reported the hydrolytic and inactivation actions of metallo-proteinases, which belong to the metzincin class (peptidase family M8), in triggering the immunoglobulin G, in addition to its role in the inactivation of the C3b factor of the complement cascade. Furthermore, M8 contributed to several functions that include the adhesion and internalisation of the parasite in macrophages, the induction of a Th1 profile response, the downregulation of the expressions of Gp63, iNOS and IL-12, and the cleavage of NF-κB [139,140,141]. Regarding their role at the species level, M8 might influence the proliferation of NK cells in humans during the infection by *L. (L.) major*, in addition to its role on cleavage CD4 glycoprotein on human T cells [142]. Meanwhile, M8 interferes with the signalling cascades and transcription factors in murine macrophages infected with *L. (L.) mexicana*. Revising the available literature, previous reports documented the major role played by oligopeptidase B (OPB) of *Leishmania* as serine proteinases (SPs) in parasite virulence and immune response against the infection, in addition to their association with signal peptidase, metacaspase, and maturase-like activity, confirming their essential functions in parasite physiology [107,143]. In this concern, OPB facilitates the establishment of the infection of murine macrophages by *L. (L.) donovani* [144], while it is associated with maintaining the infection of murine macrophage in *L. (L.) major* [145]. A recent study proposed that promastigotes of *L. (V.) braziliensis* express SPs that contribute to the maintenance of this parasite’s lifestyle at physiological pH and the out membrane of the parasite [134]. Taken together, odorant-binding protein (OBP) regulates the degradation of enolase–plasminogen complexes of the parasite, immune evasion, and disease pathology [144].

In accordance with Glycoprotein 63 (Gp63) or leishmanolysin, it is a major surface protease antigenic glycoprotein that involves parasite–host interactions and parasite virulence through the attachment of the parasite to macrophages [146]. It was originally identified as a 63–68 kDa glycoprotein anchored in the membrane via a GPI anchor [59,147,148,149,150]. Gp63 has been identified in both stages of all major pathogenic species of *Leishmania*, but it is mostly found on the surface membrane of promastigotes, where it is endowed with proteolytic activity [59,147,148,151,152]. Gp63 is involved in the modulation of the host response against the infection, but it is downregulated in the amastigote phase and this reduced expression is compensated by the absence of LPG on the surface of amastigotes, enabling Gp63 from the modulation of the host response against the infection [153]. It should be stressed that Gp63 plays different roles depending on the parasite stage. In accordance with its functions in the promastigote phase, Gp63 was reported to cleave C3b into iC3b in *L. major* and *L. amazonensis*, which protect the parasite from complement-mediated lysis [154]. Furthermore, iC3b could play an opsonin-like action which helps in establishing the interaction between the parasite and macrophages, resulting in its internalisation. Gp63 also interacts with fibronectin receptor (FR) like receptors which enhance the adherence of the parasite to macrophages [155]. Interestingly, a previous study revealed that Gp63 resulted in the degradation of various proteins from the extracellular matrix of subcutaneous tissue when they become in contact with *L. mexicana* promastigotes, which in turns alters the macrophages functions and favours the parasite survival [156]. Similarly, previous reports documented the protective action of Gp63 in *L. mexicana* that was expressed by the protection of proteins entrapped in liposomes from phagolysosomal degradation when coated with Gp63 [157]. In addition, the Gp63 molecule was reported to protect the amastigote phase of the parasite from the harsh environment of macrophages [158].Thus, the co-localisation of GP63 with the macrophage lipid raft microdomains during the infection represents one of these mechanisms [159]. Gp63 is also associated with the hydrolysis of myristoylated alanine-rich C kinasesubstrate (MARCKS) and their related proteins (MRPs), which represent substrates in macrophages [160]. In addition, Gp63 is associated with protein kinase C(PKC) critical serine/threonine kinases which are involved in cell proliferation, differentiation and apoptosis [160]. Another study revealed the critical role played by *Leishmania* Gp63 in the activation and regulation of three major protein tyrosine phosphatase (PTPs), namely SHP-1, PTP1B and TCPTP, involved in JAK2/STAT1a pathway, that represent a major player in Interferon gamma (IFNγ)-mediated signalling and their critical roles in the regulation of NO, targeting parasite survival [161,162]. Importantly, Gp63 demonstrated profound impacts on several host cell transcription factors and translational systems as shown in *L. major*, whereas Gp63 manipulated the host translational system by the cleaving of mTOR, leading to 4E-BP1 dephosphorylation, and favouring the parasite survival [163]. In addition to their association with the modification of cytokine profiles, these reported actions of Gp63 represent the major escape mechanisms of *Leishmania* from the killing mechanisms of host macrophages. Moreover, Gp63 involves the action of other phagocytic and non-phagocytic cells that are involved in the establishment of infection, e.g., they influence the release of IFNγ by natural killer (NK) cells, affecting the Th1 immune response against the parasite [142]. In addition to its correlation with infectivity and host–parasite interactions [142], the effects of Gp63 include influencing host cell signalling mechanisms and their related functions [164], the cleavage and degradation of various kinases and transcription factors, which exhibit proteolytic properties via the inhibition of the relevant enzymes [142,148,157,165,166,167]. Furthermore, the proteolytic activity of such metalloenzymes on the surface of these parasites protects their membranes from cytolytic damage during their survival, as well as the differentiation and multiplication in the phagolysosomes of macrophages [157]. More interestingly, Gp63 is also immunogenic, and therefore, it has been used as an antigen for immunodiagnosis and immunoprophylaxis [168,169,170]. It should be kept in mind that some recent reports revealed that Gp63 is highly polymorphic, even among parasites in the same endemic area; however, it seems that the functional domains are conserved in the host environment [171]. It is noteworthy to mention that the role played by Gp63 in the pathogenesis of *Leishmania* was confirmed through the deletion of the entire 20 kb region that contains the seven leishmanolysin genes (Gp63 genes 1–7). It seems that the resulting expressed promastigote forms of Gp63 1–6 genes played no role in nutrient utilisation in the early stages of parasite development in the sand flies, while these promastigotes showed a marked increase in their sensitivity to complement-mediated lysis besides a marked delay in the development of the lesions in murine models, revealing their major protective role against complement-mediated lysis [172,173]. Taken together, it seems that Gp63 play a vital role as virulence factor during *Leishmania* pathogenesis.

### 2.7. Nucleotidases

Nucleotidases are a group of membrane-anchored proteins facing the extracellular milieu. Several studies have reported that 5-nucleotidase and 3-nucleotidase/nuclease are involved in parasite nutrition through the generation of nucleotides and phosphate from nucleic acids, in addition to their role in the establishment of infection in some trypanosomatids [174,175,176]. Simulating many parasites, *Leishmania* is unable to engage in de novo purine biosynthesis, and therefore, different species of the genus *Leishmania* have developed a well organised pathway specialised in extracellular purine salvaging for survival [176,177,178]. Ecto-nucleotidases are enzymes involved in the hydrolysis of extracellular nucleotide tri- and/or di-phosphate into monophosphate products, which are subsequently hydrolysed into adenosine and play an important role in purinergic signalling; therefore, they might be capable of modulating the host immune system, which explains the direct relationship between the ability to hydrolyse nucleotides and the ability to sustain infection [174,178,179]. Furthermore, the ecto-nucleotidases are involved in the generation of nucleosides that are able to cross plasma membrane via specialised transporters (purine receptors), and they allow *Leishmania* to escape killing via neutrophil extracellular traps; therefore, they participate in parasite infectivity and the clinical outcome of the infection [174,175,179,180]. Likewise, ecto-nucleoside triphosphate diphosphohydrolase (E-NTPDase) in parasites can act as an adhesion protein during the early stages of infection, contributing to the modulation of the macrophage signalling pathways and the intracellular survival of *Leishmania* [181]. These enzymes require alkaline pH to work properly, and this may suggest that they serve such functions for metacyclic and procyclic promastigotes rather than amastigotes [59,174,179,182].

### 2.8. Heat-Shock Proteins (HSPs)

Heat-shock proteins (HSPs) are molecules with different molecular weights that act as chaperons in peptide folding; under certain stress conditions, such as temperature shock, these molecules will increase and bind to the cellular proteins to sustain the folding of the proteins [183]. The members of the family Trypanosomatidae express highly conserved members of HSP families. *Leishmania* species possess a full set of HSPs that play an important role in the biphasic life cycle of the parasite [184,185]. These proteins are highly abundant in both stages of the parasite. It is noteworthy to mention that HSP100 null mutants failed to establish the experimental infection by *L. major* and targeted the proliferation and survival of *L. donovani* inside murine macrophages [186,187]. This action of HSP 100 could be attributed to its immune modulatory action associated with sorting proteins into exosomes [188]. However, it should be stressed that HSP100 are not involved in the thermotolerance in *L. major* and *L. donovani*. In addition, HSP83, a homolog of HSP90, is a regulatory element in the 3′ untranslated region (UTR) of Hsp83 which controls the translation of Hsp83 in a temperature-sensitive manner [189]. This category of protein plays an important role in the folding, assembly, intracellular localisation, secretion, regulation, stabilisation and degradation of other proteins [190]. HSP90 and its co-chaperones represent integral parts of the signal transduction pathways during the life cycle of several species of *Leishmania*, e.g., *L. donovani*, in addition to their crucial role in a stage-specific phosphorylation process [191,192,193]. Furthermore, the post-translational modification of HSP90 and its co-chaperones are associated to *Leishmania* viability [194]. Taken into consideration, HSP 70 and HSP 40 are diverse in *Leishmania*. However, few studies have demonstrated their possible roles during the parasite life cycle. It is noteworthy to state that HSP 70 and HSP 40 are members of foldosome, together with HSP 90, and several co-chaperones, e.g., P23 (Sba1) and Sti-1 (HOP), that contribute to the process of activation and maturation of the essential proteins [184]. In accordance with the HSP23 of *L. donovonai*, it has been considered an important virulence factor necessary for parasite survival at mammalian host. This function was confirmed in HSP23-null mutants, which became non-infectious to primary macrophages in vitro [195,196]. Taking all these facts together, HSPs may play major roles in parasite differentiation/survival during infection with *Leishmania* [197]. These proteins are crucial for temperature-induced differentiation from the promastigote to the amastigote stage, in addition to their role in intracellular survival within the mammalian host [195,196]. An increase in virulence has been briefly reported with heat shocked promastigotes [59]. Furthermore, these proteins have been implicated in the induction of human T-cell and protective immune responses because they enhance dendritic cells to produce several inflammatory cytokines. Clearly, HSPs are involved in antigen processing and presentation pathways, in addition to their role in the development of tissue damage in strong hypersensitive reactions cases [198]. In conclusion, HSPs modification might play a pivotal role in parasite survival at the mammalian host temperature, and as a consequence, in the development of parasitic resistance to chemotherapy [196,199].

### 2.9. Transporters

Several transmembrane transporters have been reported in all organisms, ranging from bacteria to mammals, and they belong to the family of ABC transporters (traffic ATPases) [200,201]. The ATP binding domains of ABC transporters are around 200 amino acids long. *Leishmania* encodes many putative membrane transporters because it possesses a transport system for carbohydrate, glucose, folate, proline ribose, nucleobase, nucleosides, amino acid, and cation or proton transporting ATPase, which seems extremely important in the promastigote stage of the life cycle [202,203,204]. These systems are crucial for parasite homeostasis, ion acquisition and the transportation of certain essential nutrients, such as lipid movements across the plasma membrane, and therefore, any alteration in these mechanisms will affect parasite homeostasis combined with their importance in the viability and infectivity of the disease-causing amastigote stages of *L. mexicana*. Furthermore, their activity might affect vesicle trafficking, and therefore, could be a key to developing novel antileishmanial drugs and understanding the mechanisms underlying drug resistance [59,205,206,207,208].

## 3. Conclusions

**Figure 2:**
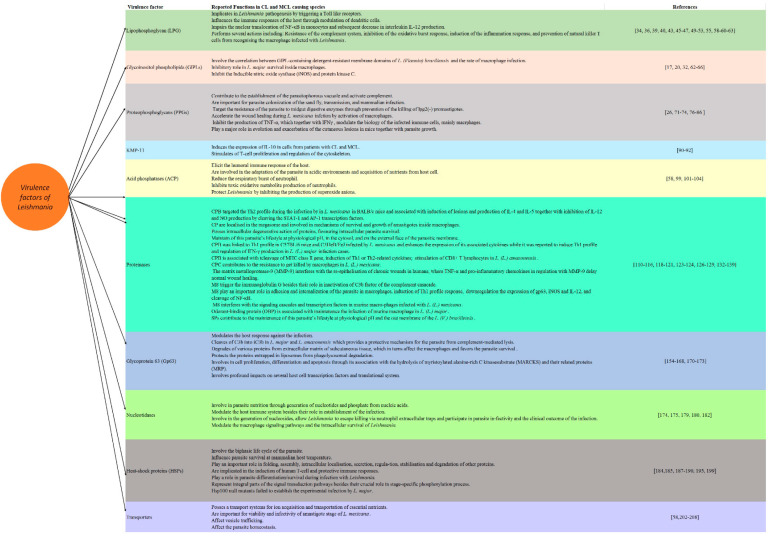
The virulence factors of cutaneous leishmaniasis (CL)/mucocutaneous leishmaniasis (MCL) causing species of *Leishmania* and their reported function.

**Figure 3:**
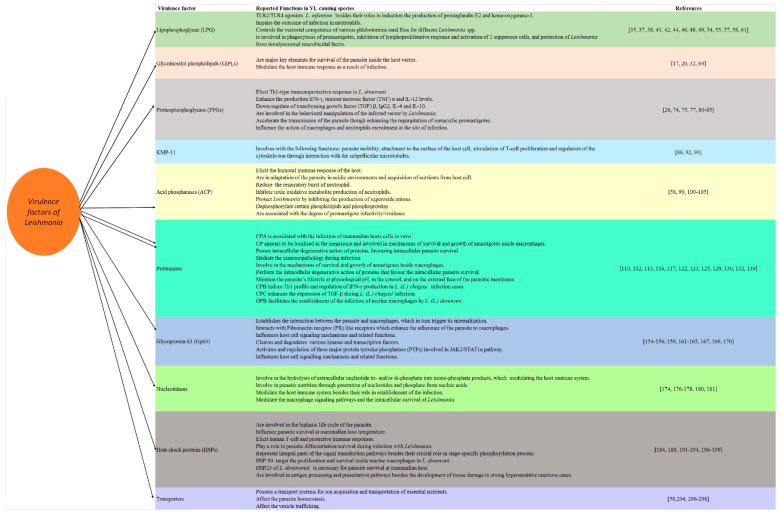
The virulence factors of visceral leishmaniasis (VL) causing species of *Leishmania* and their reported functions.

*Leishmania* possesses the ability to persist in host cells by modulating the host immune system via several mechanisms, including the induction of immunosuppression or the modification of the chemokine profiles of the host. The pathogenesis of leishmaniasis is highly variable depending on several factors, including the infecting species and its virulence factors, as well as the host, which determine the course of the disease. In addition to their important role in survival in the host cells, the parasites’ virulence factors are crucial for seeking novel drugs, drug targets and vaccine preparation (Figure 2 and Figure 3). Further future research to explore the virulence factors of various *Leishmania* species seems interesting for establishing a better understanding of the pathogenesis of the disease that would be helpful in designing a novel vaccine for combating the disease.

## Data Availability

Not applicable.

## References

[1] Clem A. (2010). A current perspective on leishmaniasis. J. Glob. Infect. Dis..

[2] Romero H.D., Lde A.S., Silva-Vergara M.L., Rodrigues V., Costa R.T., Guimaraes S.F., Alecrim W., Moraes-Souza H., Prata A. (2009). Comparative study of serologic tests for the diagnosis of asymptomatic visceral leishmaniasis in an endemic area. Am. J. Trop. Med. Hyg..

[3] WHO (2010). Control of the leishmaniases. World Health Organ. Tech. Rep. Ser..

[4] Lozano R., Naghavi M., Foreman K., Lim S., Shibuya K., Aboyans V., Abraham J., Adair T., Aggarwal R., Ahn S.Y. (2012). Global and regional mortality from 235 causes of death for 20 age groups in 1990 and 2010: A systematic analysis for the Global Burden of Disease Study 2010. Lancet.

[5] Barrett M.P., Croft S.L. (2012). Management of trypanosomiasis and leishmaniasis. Br. Med. Bull..

[6] Elmahallawy E.K., Agil A. (2015). Treatment of leishmaniasis: A review and assessment of recent research. Curr. Pharm. Dis..

[7] Elmahallawy E.K., Martinez A.S., Rodriguez-Granger J., Hoyos-Mallecot Y., Agil A., Mari J.M.N., Fernandez J.G. (2014). Diagnosis of leishmaniasis. J. Infect. Dev. Ctries..

[8] Herwaldt B.L. (1999). Leishmaniasis. Lancet.

[9] Desjeux P. (1992). Human leishmaniases: Epidemiology and public health aspects. World Health Stat. Q. Rapp. Trimest. Stat. Sanit. Mond..

[10] Desjeux P. (2004). Leishmaniasis: Current situation and new perspectives. Comp. Immunol. Microbiol. Infect. Dis..

[11] Chang K.P., Bray R.C. (1985). Human Parasitic Diseases: Leishmaniasis.

[12] Chappuis F., Sundar S., Hailu A., Ghalib H., Rijal S., Peeling R.W., Alvar J., Boelaert M. (2007). Visceral leishmaniasis: What are the needs for diagnosis, treatment and control?. Nat. Rev. Microbiol.

[13] Okwor I., Uzonna J. (2008). Persistent parasites and immunologic memory in cutaneous leishmaniasis: Implications for vaccine designs and vaccination strategies. Immunol. Res..

[14] Vanloubbeeck Y., Jones D.E. (2004). The immunology of Leishmania infection and the implications for vaccine development. Ann. N. Y. Acad. Sci..

[15] Contreras I., Gomez M.A., Nguyen O., Shio M.T., McMaster R.W., Olivier M. (2010). Leishmania-induced inactivation of the macrophage transcription factor AP-1 is mediated by the parasite metalloprotease GP63. PLoS Pathog..

[16] Shio M.T., Hassani K., Isnard A., Ralph B., Contreras I., Gomez M.A., Abu-Dayyeh I., Olivier M. (2012). Host cell signalling and leishmania mechanisms of evasion. J. Trop. Med..

[17] Brandonisio O., Panaro M.A., Sisto M., Acquafredda A., Fumarola L., Leogrande D. (2000). Interactions between Leishmania parasites and host cells. Parassitologia.

[18] Olivier M., Atayde V.D., Isnard A., Hassani K., Shio M.T. (2012). Leishmania virulence factors: Focus on the metalloprotease GP63. Microbes Infect..

[19] Naderer T., Vince J.E., McConville M.J. (2004). Surface determinants of Leishmania parasites and their role in infectivity in the mammalian host. Curr. Mol. Med..

[20] Silva-Almeida M., Pereira B.A., Ribeiro-Guimaraes M.L., Alves C.R. (2012). Proteinases as virulence factors in Leishmania spp. infection in mammals. Parasit Vectors.

[21] Buxbaum L.U. (2013). Leishmania mexicana infection induces IgG to parasite surface glycoinositol phospholipids that can induce IL-10 in mice and humans. PLoS Negl. Trop. Dis..

[22] Zufferey R., Allen S., Barron T., Sullivan D.R., Denny P.W., Almeida I.C., Smith D.F., Turco S.J., Ferguson M.A., Beverley S.M. (2003). Ether phospholipids and glycosylinositolphospholipids are not required for amastigote virulence or for inhibition of macrophage activation by Leishmania major. J. Biol. Chem..

[23] Matlashewski G. (2001). Leishmania infection and virulence. Med. Microbiol. Immunol..

[24] Dostalova A., Volf P. (2012). Leishmania development in sand flies: Parasite-vector interactions overview. Parasites Vectors.

[25] Spath G.F., Epstein L., Leader B., Singer S.M., Avila H.A., Turco S.J., Beverley S.M. (2000). Lipophosphoglycan is a virulence factor distinct from related glycoconjugates in the protozoan parasite Leishmania major. Proc. Natl. Acad. Sci. USA.

[26] Rogers M.E. (2012). The role of leishmania proteophosphoglycans in sand fly transmission and infection of the Mammalian host. Front. Microbiol..

[27] Campos M.P., Figueiredo F.B., Morgado F.N., Renzetti A.R.d.S., de Souza S.M.M., Pereira S.A., Rodrigues-Da-Silva R.N., Lima-Junior J.D.C., De Luca P.M. (2018). Leishmania infantum Virulence Factor A2 Protein: Linear B-Cell Epitope Mapping and Identification of Three Main Linear B-Cell Epitopes in Vaccinated and Naturally Infected Dogs. Front. Immunol..

[28] Guha R., Das S., Ghosh J., Naskar K., Mandala A., Sundar S., Dujardin J.C., Roy S. (2013). Heterologous priming-boosting with DNA and vaccinia virus expressing kinetoplastid membrane protein-11 induces potent cellular immune response and confers protection against infection with antimony resistant and sensitive strains of Leishmania (Leishmania) donovani. Vaccine.

[29] Proudfoot L., O’Donnell C.A., Liew F.Y. (1995). Glycoinositolphospholipids of Leishmania major inhibit nitric oxide synthesis and reduce leishmanicidal activity in murine macrophages. Eur. J. Immunol..

[30] Chang K.P., Reed S.G., McGwire B.S., Soong L. (2003). Leishmania model for microbial virulence: The relevance of parasite multiplication and pathoantigenicity. Acta Trop..

[31] Chang K.P., McGwire B.S. (2002). Molecular determinants and regulation of Leishmania virulence. Kinetoplastid Biol. Dis..

[32] da Pires S.F., Fialho L.C., Silva S.O., Melo M.N., de Souza C.C., Tafuri W.L., Romero O.B., de Andrade H.M. (2014). Identification of virulence factors in Leishmania infantum strains by a proteomic approach. J. Proteome Res..

[33] Forestier C.L., Gao Q., Boons G.J. (2014). Leishmania lipophosphoglycan: How to establish structure-activity relationships for this highly complex and multifunctional glycoconjugate?. Front. Cell. Infect. Microbiol..

[34] Lodge R., Descoteaux A. (2005). Leishmania donovani promastigotes induce periphagosomal F-actin accumulation through retention of the GTPase Cdc42. Cell Microbiol..

[35] de Assis R.R., Ibraim I.C., Nogueira P.M., Soares R.P., Turco S.J. (2012). Glycoconjugates in New World species of Leishmania: Polymorphisms in lipophosphoglycan and glycoinositolphospholipids and interaction with hosts. Biochim. Biophys. Acta.

[36] Sacks D.L., Pimenta P., McConville M.J., Schneider P., Turco S.J. (1995). Stage-specific binding of Leishmania donovani to the sand fly vector midgut is regulated by conformational changes in the abundant surface lipophosphoglycan. J. Exp. Med..

[37] Sacks D.L., Brodin T.N., Turco S.J. (1990). Developmental modification of the lipophosphoglycan from Leishmania major promastigotes during metacyclogenesis. Mol. Biochem. Parasitol..

[38] Soares R.P., Macedo M.E., Ropert C., Gontijo N.F., Almeida I.C., Gazzinelli R.T., Pimenta P.F., Turco S.J. (2002). Leishmania chagasi: Lipophosphoglycan characterization and binding to the midgut of the sand fly vector Lutzomyia longipalpis. Mol. Biochem. Parasitol..

[39] Coelho-Finamore J., Freitas V., Assis R., Melo M., Novozhilova N., Secundino N., Pimenta P., Turco S., Soares R. (2011). Leishmania infantum: Lipophosphoglycan intraspecific variation and interaction with vertebrate and invertebrate hosts. Int. J. Parasitol..

[40] Volf P., Nogueira P.M., Myskova J., Turco S.J., Soares R.P. (2014). Structural comparison of lipophosphoglycan from Leishmania turanica and L. major, two species transmitted by Phlebotomus papatasi. Parasitol. Int..

[41] Dobson D.E., Scholtes L.D., Myler P.J., Turco S.J., Beverley S.M. (2006). Genomic organization and expression of the expanded SCG/L/R gene family of Leishmania major: Internal clusters and telomeric localization of SCGs mediating species-specific LPG modifications. Mol. Biochem. Parasitol..

[42] Cardoso C.A., Araujo G.V., Sandoval C.M., Nogueira P.M., Zúniga C., Sosa-Ochoa W.H., Laurenti M.D., Soares R.P. (2020). Lipophosphoglycans from dermotropic Leishmania infantum are more pro-inflammatory than those from viscerotropic strains. Memórias Instituto Oswaldo Cruz.

[43] Ibraim I.C., de Assis R.R., Pessoa N.L., Campos M.A., Melo M.N., Turco S.J., Soares R.P. (2013). Two biochemically distinct lipophosphoglycans from Leishmania braziliensis and Leishmania infantum trigger different innate immune responses in murine macrophages. Parasites Vectors.

[44] Nogueira P.M., Assis R.R., Torrecilhas A.C., Saraiva E.M., Pessoa N.L., Campos M.A., Marialva E.F., Ríos-Velasquez C.M., Pessoa F.A., Secundino N.F. (2016). Lipophosphoglycans from Leishmania amazonensis strains display immunomodulatory properties via TLR4 and do not affect sand fly infection. PLoS Negl. Trop. Dis..

[45] McConville M.J., Blackwell J.M. (1991). Developmental changes in the glycosylated phosphatidylinositols of Leishmania donovani. Characterization of the promastigote and amastigote glycolipids. J. Biol. Chem..

[46] Bahr V., Stierhof Y.D., Ilg T., Demar M., Quinten M., Overath P. (1993). Expression of lipophosphoglycan, high-molecular weight phosphoglycan and glycoprotein 63 in promastigotes and amastigotes of Leishmania mexicana. Mol. Biochem. Parasitol..

[47] Amer A.O., Swanson M.S. (2002). A phagosome of one’s own: A microbial guide to life in the macrophage. Curr. Opin. Microbiol..

[48] Spath G.F., Garraway L.A., Turco S.J., Beverley S.M. (2003). The role(s) of lipophosphoglycan (LPG) in the establishment of Leishmania major infections in mammalian hosts. Proc. Natl. Acad. Sci. USA.

[49] Lima J.B., Araujo-Santos T., Lazaro-Souza M., Carneiro A.B., Ibraim I.C., Jesus-Santos F.H., Luz N.F., Pontes S.M., Entringer P.F., Descoteaux A. (2017). Leishmania infantum lipophosphoglycan induced-Prostaglandin E2 production in association with PPAR-gamma expression via activation of Toll like receptors-1 and 2. Sci. Rep..

[50] Handman E., McConville M.J., Goding J.W. (1987). Carbohydrate antigens as possible parasite vaccines A case for the Leishmania glycolipid. Immunol. Today.

[51] Svarovska A., Ant T.H., Seblova V., Jecna L., Beverley S.M., Volf P. (2010). Leishmania major glycosylation mutants require phosphoglycans (lpg2-) but not lipophosphoglycan (lpg1-) for survival in permissive sand fly vectors. PLoS Negl. Trop. Dis..

[52] Argueta-Donohue J., Carrillo N., Valdes-Reyes L., Zentella A., Aguirre-Garcia M., Becker I., Gutierrez-Kobeh L. (2008). Leishmania mexicana: Participation of NF-kappaB in the differential production of IL-12 in dendritic cells and monocytes induced by lipophosphoglycan (LPG). Exp. Parasitol..

[53] Von Stebut E. (2007). Immunology of cutaneous leishmaniasis: The role of mast cells, phagocytes and dendritic cells for protective immunity. Eur. J. Dermatol..

[54] Carrada G., Caneda C., Salaiza N., Delgado J., Ruiz A., Sanchez B., Gutierrez-Kobeh L., Aguirre M., Becker I. (2007). Monocyte cytokine and costimulatory molecule expression in patients infected with Leishmania mexicana. Parasite Immunol..

[55] Luz N.F., Andrade B.B., Feijó D.F., Araújo-Santos T., Carvalho G.Q., Andrade D., Abánades D.R., Melo E.V., Silva A.M., Brodskyn C.I. (2012). Heme oxygenase-1 promotes the persistence of Leishmania chagasi infection. J. Immunol..

[56] Liu D., Kebaier C., Pakpour N., Capul A.A., Beverley S.M., Scott P., Uzonna J.E. (2009). Leishmania major phosphoglycans influence the host early immune response by modulating dendritic cell functions. Infect. Immun..

[57] Pimenta P.F., Saraiva E.M., Rowton E., Modi G.B., Garraway L.A., Beverley S.M., Turco S.J., Sacks D.L. (1994). Evidence that the vectorial competence of phlebotomine sand flies for different species of Leishmania is controlled by structural polymorphisms in the surface lipophosphoglycan. Proc. Natl. Acad. Sci. USA.

[58] Jesus-Santos F.H., Lobo-Silva J., Ramos P.I.P., Descoteaux A., Lima J.B., Borges V.M., Farias L.P. (2020). LPG2 gene duplication in Leishmania infantum: A case for CRISPR-Cas9 gene editing. Front. Cell. Infect. Microbiol..

[59] Chang K.P., Chaudhuri G., Fong D. (1990). Molecular determinants of Leishmania virulence. Annu. Rev. Microbiol..

[60] Da Silva R.P., Hall B.F., Joiner K.A., Sacks D.L. (1989). CR1, the C3b receptor, mediates binding of infective Leishmania major metacyclic promastigotes to human macrophages. J. Immunol..

[61] Ivens A.C., Peacock C.S., Worthey E.A., Murphy L., Aggarwal G., Berriman M., Sisk E., Rajandream M.A., Adlem E., Aert R. (2005). The genome of the kinetoplastid parasite, Leishmania major. Science.

[62] McConville M.J., Bacic A. (1989). A family of glycoinositol phospholipids from Leishmania major. Isolation, characterization, and antigenicity. J. Biol. Chem..

[63] Yoneyama K.A., Tanaka A.K., Silveira T.G., Takahashi H.K., Straus A.H. (2006). Characterization of Leishmania (Viannia) braziliensis membrane microdomains, and their role in macrophage infectivity. J. Lipid Res..

[64] Broermann L., Heidenreich W. (1992). Malaria tropica and pregnancy. Geburtshilfe Und Frauenheilkd.

[65] Passero L.F., Assis R.R., da Silva T.N., Nogueira P.M., Macedo D.H., Pessoa N.L., Campos M.A., Laurenti M.D., Soares R.P. (2015). Differential modulation of macrophage response elicited by glycoinositolphospholipids and lipophosphoglycan from Leishmania (Viannia) shawi. Parasitol. Int..

[66] Paranaíba L.F., de Assis R.R., Nogueira P.M., Torrecilhas A.C., Campos J.H., de Silveira A.C.O., Martins-Filho O.A., Pessoa N.L., Campos M.A., Parreiras P.M. (2015). Leishmania enriettii: Biochemical characterisation of lipophosphoglycans (LPGs) and glycoinositolphospholipids (GIPLs) and infectivity to Cavia porcellus. Parasites Vectors.

[67] Ilg T., Montgomery J., Stierhof Y.D., Handman E. (1999). Molecular cloning and characterization of a novel repeat-containing Leishmania major gene, ppg1, that encodes a membrane-associated form of proteophosphoglycan with a putative glycosylphosphatidylinositol anchor. J. Biol. Chem..

[68] Ilg T. (2000). Proteophosphoglycans of Leishmania. Parasitol. Today.

[69] Klein C., Gopfert U., Goehring N., Stierhof Y.D., Ilg T. (1999). Proteophosphoglycans of Leishmania mexicana. Identification, purification, structural and ultrastructural characterization of the secreted promastigote proteophosphoglycan pPPG2, a stage-specific glycoisoform of amastigote aPPG. Biochem. J..

[70] Piani A., Ilg T., Elefanty A.G., Curtis J., Handman E. (1999). Leishmania major proteophosphoglycan is expressed by amastigotes and has an immunomodulatory effect on macrophage function. Microbes Infect..

[71] Aebischer T., Harbecke D., Ilg T. (1999). Proteophosphoglycan, a major secreted product of intracellular Leishmania mexicana amastigotes, is a poor B-cell antigen and does not elicit a specific conventional CD4+ T-cell response. Infect. Immun..

[72] Peters C., Kawakami M., Kaul M., Ilg T., Overath P., Aebischer T. (1997). Secreted proteophosphoglycan of Leishmania mexicana amastigotes activates complement by triggering the mannan binding lectin pathway. Eur. J. Immunol..

[73] Peters C., Stierhof Y.D., Ilg T. (1997). Proteophosphoglycan secreted by Leishmania mexicana amastigotes causes vacuole formation in macrophages. Infect. Immun..

[74] Duque G.A., Jardim A., Gagnon E., Fukuda M., Descoteaux A. (2019). The host cell secretory pathway mediates the export of Leishmania virulence factors out of the parasitophorous vacuole. PLoS Pathog..

[75] Samant M., Gupta R., Kumari S., Misra P., Khare P., Kushawaha P.K., Sahasrabuddhe A.A., Dube A. (2009). Immunization with the DNA-encoding N-terminal domain of proteophosphoglycan of Leishmania donovani generates Th1-type immunoprotective response against experimental visceral leishmaniasis. J. Immunol.

[76] Rogers M.E., Ilg T., Nikolaev A.V., Ferguson M.A., Bates P.A. (2004). Transmission of cutaneous leishmaniasis by sand flies is enhanced by regurgitation of fPPG. Nature.

[77] Rogers M.E., Bates P.A. (2007). Leishmania manipulation of sand fly feeding behavior results in enhanced transmission. PLoS Pathog..

[78] Secundino N., Kimblin N., Peters N.C., Lawyer P., Capul A.A., Beverley S.M., Turco S.J., Sacks D. (2010). Proteophosphoglycan confers resistance of Leishmania major to midgut digestive enzymes induced by blood feeding in vector sand flies. Cell. Microbiol..

[79] Giraud E., Lestinova T., Derrick T., Martin O., Dillon R.J., Volf P., Műller I., Bates P.A., Rogers M.E. (2018). Leishmania proteophosphoglycans regurgitated from infected sand flies accelerate dermal wound repair and exacerbate leishmaniasis via insulin-like growth factor 1-dependent signalling. PLoS Pathog..

[80] Stierhof Y.-D., Bates P.A., Jacobson R.L., Rogers M.E., Schlein Y., Handman E., Ilg T. (1999). Filamentous proteophosphoglycan secreted by Leishmania promastigotes forms gel-like three-dimensional networks that obstruct the digestive tract of infected sandfly vectors. Eur. J. Cell Biol..

[81] Rogers M., Kropf P., Choi B.-S., Dillon R., Podinovskaia M., Bates P., Müller I. (2009). Proteophosophoglycans regurgitated by Leishmania-infected sand flies target the L-arginine metabolism of host macrophages to promote parasite survival. PLoS Pathog..

[82] Sacks D., Kamhawi S. (2001). Molecular aspects of parasite-vector and vector-host interactions in leishmaniasis. Annu. Rev. Microbiol..

[83] Peters N.C., Egen J.G., Secundino N., Debrabant A., Kimblin N., Kamhawi S., Lawyer P., Fay M.P., Germain R.N., Sacks D. (2008). In vivo imaging reveals an essential role for neutrophils in leishmaniasis transmitted by sand flies. Science.

[84] Bogdan C. (2001). Nitric oxide and the immune response. Nat. Immunol..

[85] Kropf P., Fuentes J.M., Fähnrich E., Arpa L., Herath S., Weber V., Soler G., Celada A., Modolell M., Müller I. (2005). Arginase and polyamine synthesis are key factors in the regulation of experimental leishmaniasis in vivo. FASEB J..

[86] Giraud E., Svobodová M., Müller I., Volf P., Rogers M. (2019). Promastigote secretory gel from natural and unnatural sand fly vectors exacerbate Leishmania major and Leishmania tropica cutaneous leishmaniasis in mice. Parasitology.

[87] Jardim A., Funk V., Caprioli R.M., Olafson R.W. (1995). Isolation and structural characterization of the Leishmania donovani kinetoplastid membrane protein-11, a major immunoreactive membrane glycoprotein. Biochem. J..

[88] Tolson D.L., Jardim A., Schnur L.F., Stebeck C., Tuckey C., Beecroft R.P., Teh H.S., Olafson R.W., Pearson T.W. (1994). The kinetoplastid membrane protein 11 of Leishmania donovani and African trypanosomes is a potent stimulator of T-lymphocyte proliferation. Infect. Immun..

[89] Moody S.F. (1993). Molecular variation in Leishmania. Acta Trop..

[90] Matos D.C., Faccioli L.A., Cysne-Finkelstein L., Luca P.M., Corte-Real S., Armoa G.R., Lemes E.M., Decote-Ricardo D., Mendonca S.C. (2010). Kinetoplastid membrane protein-11 is present in promastigotes and amastigotes of Leishmania amazonensis and its surface expression increases during metacyclogenesis. Memórias Instituto Oswaldo Cruz.

[91] Carvalho L.P., Passos S., Dutra W.O., Soto M., Alonso C., Gollob K.J., Carvalho E.M., de Jesus A.R. (2005). Effect of LACK and KMP11 on IFN-gamma production by peripheral blood mononuclear cells from cutaneous and mucosal leishmaniasis patients. Scand. J. Immunol..

[92] Skeiky Y.A., Guderian J.A., Benson D.R., Bacelar O., Carvalho E.M., Kubin M., Badaro R., Trinchieri G., Reed S.G. (1995). A recombinant Leishmania antigen that stimulates human peripheral blood mononuclear cells to express a Th1-type cytokine profile and to produce interleukin 12. J. Exp. Med..

[93] Mukhopadhyay S., Sen P., Majumder H.K., Roy S. (1998). Reduced expression of lipophosphoglycan (LPG) and kinetoplastid membrane protein (KMP)-11 in Leishmania donovani promastigotes in axenic culture. J. Parasitol..

[94] Pfeiffer Y., Tal E., Sulman F.G. (1978). Heat-induced migraine and its treatment. Harefuah.

[95] Bates P.A., Dwyer D.M. (1987). Biosynthesis and secretion of acid phosphatase by Leishmania donovani promastigotes. Mol. Biochem. Parasitol..

[96] Freitas-Mesquita A.L., Fonseca-de-Souza A.L., Meyer-Fernandes J.R. (2014). Leishmania amazonensis: Characterization of an ecto-pyrophosphatase activity. Exp. Parasitol..

[97] Ilg T., Stierhof Y.D., Etges R., Adrian M., Harbecke D., Overath P. (1991). Secreted acid phosphatase of Leishmania mexicana: A filamentous phosphoglycoprotein polymer. Proc. Natl. Acad. Sci. USA.

[98] Stierhof Y.D., Wiese M., Ilg T., Overath P., Haner M., Aebi U. (1998). Structure of a filamentous phosphoglycoprotein polymer: The secreted acid phosphatase of Leishmania mexicana. J. Mol. Biol..

[99] Glew R.H., Saha A.K., Das S., Remaley A.T. (1988). Biochemistry of the Leishmania species. Microbiol. Rev..

[100] Remaley A.T., Glew R.H., Kuhns D.B., Basford R.E., Waggoner A.S., Ernst L.A., Pope M. (1985). Leishmania donovani: Surface membrane acid phosphatase blocks neutrophil oxidative metabolite production. Exp. Parasitol..

[101] Kane M.M., Mosser D.M. (2000). Leishmania parasites and their ploys to disrupt macrophage activation. Curr. Opin. Hematol..

[102] Stafford J.L., Neumann N.F., Belosevic M. (2002). Macrophage-mediated innate host defense against protozoan parasites. Crit. Rev. Microbiol..

[103] Saha A.K., Das S., Glew R.H., Gottlieb M. (1985). Resistance of leishmanial phosphatases to inactivation by oxygen metabolites. J. Clin. Microbiol..

[104] Ellis S.L., Shakarian A.M., Dwyer D.M. (1998). Leishmania: Amastigotes synthesize conserved secretory acid phosphatases during human infection. Exp. Parasitol..

[105] Singla N., Khuller G.K., Vinayak V.K. (1992). Acid phosphatase activity of promastigotes of Leishmania donovani: A marker of virulence. FEMS Microbiol. Lett..

[106] Barrett A.J. (1994). Classification of peptidases. Methods Enzymol..

[107] McKerrow J.H., Caffrey C., Kelly B., Loke P.N., Sajid M. (2006). Proteases in parasitic diseases. Annu. Rev. Pathol. Mech. Dis..

[108] Rawlings N.D., Barrett A.J., Bateman A. (2010). MEROPS: The peptidase database. Nucleic Acids Res..

[109] Rawlings N.D. (2010). Peptidase inhibitors in the MEROPS database. Biochimie.

[110] North M.J., Mottram J.C., Coombs G.H. (1990). Cysteine proteinases of parasitic protozoa. Parasitol. Today.

[111] Pral E.M., Bijovsky A.T., Balanco J.M., Alfieri S.C. (1993). Leishmania mexicana: Proteinase activities and megasomes in axenically cultivated amastigote-like forms. Exp. Parasitol..

[112] Das P., Alam M.N., Paik D., Karmakar K., De T., Chakraborti T. (2013). Protease inhibitors in potential drug development for Leishmaniasis. Indian J. Biochem. Biophys..

[113] Sajid M., McKerrow J.H. (2002). Cysteine proteases of parasitic organisms. Mol. Biochem. Parasitol..

[114] Pupkis M.F., Tetley L., Coombs G.H. (1986). Leishmania mexicana: Amastigote hydrolases in unusual lysosomes. Exp. Parasitol..

[115] Rafati S., Couty-Jouve S., Alimohammadian M., Louis J. (1997). Biochemical analysis and immunogenicity of Leishmania major amastigote fractions in cutaneous leishmaniasis. Clin. Exp. Immunol..

[116] Mottram J.C., Coombs G.H., Alexander J. (2004). Cysteine peptidases as virulence factors of Leishmania. Curr. Opin. Microbiol..

[117] Denise H., Poot J., Jiménez M., Ambit A., Herrmann D.C., Vermeulen A.N., Coombs G.H., Mottram J.C. (2006). Studies on the CPA cysteine peptidase in the Leishmania infantum genome strain JPCM5. BMC Mol. Biol..

[118] Pollock K.G., McNeil K.S., Mottram J.C., Lyons R.E., Brewer J.M., Scott P., Coombs G.H., Alexander J. (2003). The Leishmania mexicana cysteine protease, CPB2. 8, induces potent Th2 responses. J. Immunol..

[119] Cameron P., McGachy A., Anderson M., Paul A., Coombs G.H., Mottram J.C., Alexander J., Plevin R. (2004). Inhibition of lipopolysaccharide-induced macrophage IL-12 production by Leishmania mexicana amastigotes: The role of cysteine peptidases and the NF-κB signaling pathway. J. Immunol..

[120] Buxbaum L.U., Denise H., Coombs G.H., Alexander J., Mottram J.C., Scott P. (2003). Cysteine protease B of Leishmania mexicana inhibits host Th1 responses and protective immunity. J. Immunol..

[121] Alexander J., Coombs G.H., Mottram J.C. (1998). Leishmania mexicana cysteine proteinase-deficient mutants have attenuated virulence for mice and potentiate a Th1 response. J. Immunol..

[122] da Pinheiro P.H.C., de Dias S.S., Eulálio K.D., Mendonça I.L., Katz S., Barbiéri C.L. (2005). Recombinant cysteine proteinase from Leishmania (Leishmania) chagasi implicated in human and dog T-cell responses. Infect. Immun..

[123] Alves C., Benévolo-de-Andrade T., Alves J., Pirmez C. (2004). Th1 and Th2 immunological profile induced by cysteine proteinase in murine leishmaniasis. Parasite Immunol..

[124] Leao S.D.S., Lang T., Prina E., Hellio R., Antoine J.-C. (1995). Intracellular Leishmania amazonensis amastigotes internalize and degrade MHC class II molecules of their host cells. J. Cell Sci..

[125] Rafati S., Fasel N., Masina S. (2003). Leishmania cysteine proteinases: From gene to subunit vaccine. Curr. Genom..

[126] Traub-Cseko Y.M., Duboise M., Boukai L.K., McMahon-Pratt D. (1993). Identification of two distinct cysteine proteinase genes of Leishmania pifanoi axenic amastigotes using the polymerase chain reaction. Mol. Biochem. Parasitol..

[127] Alves C.R., Côrte-Real S., De-Freitas M.R., Giovanni-De-Simone S. (2000). Detection of cysteine-proteinases in Leishmania amazonensis promastigotes using a cross-reactive antiserum. FEMS Microbiol. Lett..

[128] Sakanari J.A., Nadler S.A., Chan V.J., Engel J.C., Leptak C., Bouvier J. (1997). Leishmania major: Comparison of the cathepsin L-and B-like cysteine protease genes with those of other trypanosomatids. Exp. Parasitol..

[129] Caffrey C.R., Lima A.P., Steverding D. (2011). Cysteine peptidases of kinetoplastid parasites. Adv. Exp. Med. Biol..

[130] Somanna A., Mundodi V., Gedamu L. (2002). Functional Analysis of Cathepsin B-like Cysteine Proteases fromLeishmania donovani Complex: Evidence for the activation of latent transforming growth factor β. J. Biol. Chem..

[131] Frame M., Mottram J., Coombs G. (2000). Analysis of the roles of cysteine proteinases of Leishmania mexicana in the host–parasite interaction. Parasitology.

[132] Mottram J.C., Brooks D.R., Coombs G.H. (1998). Roles of cysteine proteinases of trypanosomes and Leishmania in host-parasite interactions. Curr. Opin. Microbiol..

[133] Alves C.R., Corte-Real S., Bourguignon S.C., Chaves C.S., Saraiva E.M. (2005). Leishmania amazonensis: Early proteinase activities during promastigote-amastigote differentiation in vitro. Exp. Parasitol..

[134] Santos-de-Souza R., de Castro Cortes L.M., Dos Charret K.S., Cysne-Finkelstein L., Alves C.R., Souza-Silva F. (2019). Serine Proteinases in Leishmania (Viannia) braziliensis Promastigotes Have Distinct Subcellular Distributions and Expression. Int. J. Mol. Sci..

[135] Maretti-Mira A.C., de Oliveira-Neto M.P., Da-Cruz A.M., de Oliveira M.P., Craft N., Pirmez C. (2011). Therapeutic failure in American cutaneous leishmaniasis is associated with gelatinase activity and cytokine expression. Clin. Exp. Immunol..

[136] Reiss M.J., Han Y.P., Garcia E., Goldberg M., Yu H., Garner W.L. (2010). Matrix metalloproteinase-9 delays wound healing in a murine wound model. Surgery.

[137] Campos T.M., Passos S.T., Novais F.O., Beiting D.P., Costa R.S., Queiroz A., Mosser D., Scott P., Carvalho E.M., Carvalho L.P. (2014). Matrix metalloproteinase 9 production by monocytes is enhanced by TNF and participates in the pathology of human cutaneous Leishmaniasis. PLoS Negl. Trop. Dis..

[138] Oliveira M.P., Martins V.T., Santos T.T., Lage D.P., Ramos F.F., Salles B., Costa L.E., Dias D.S., Ribeiro P.A., Schneider M.S. (2018). Small myristoylated protein-3, identified as a potential virulence factor in Leishmania amazonensis, proves to be a protective antigen against visceral leishmaniasis. Int. J. Mol. Sci..

[139] Yao C. (2010). Major surface protease of trypanosomatids: One size fits all?. Infect. Immun..

[140] Thiakaki M., Kolli B., Chang K.-P., Soteriadou K. (2006). Down-regulation of gp63 level in Leishmania amazonensis promastigotes reduces their infectivity in BALB/c mice. Microbes Infect..

[141] Gregory D.J., Godbout M., Contreras I., Forget G., Olivier M. (2008). A novel form of NF-κB is induced by Leishmania infection: Involvement in macrophage gene expression. Eur. J. Immunol..

[142] Lieke T., Nylen S., Eidsmo L., McMaster W., Mohammadi A.M., Khamesipour A., Berg L., Akuffo H. (2008). Leishmania surface protein gp63 binds directly to human natural killer cells and inhibits proliferation. Clin. Exp. Immunol..

[143] Alves C.R., de Souza R.S., dos Charret K.S., de Cortes L.M.C., de Sa-Silva M.P., Barral-Veloso L., Oliveira L.F.G., da Silva F.S. (2018). Understanding serine proteases implications on Leishmania spp lifecycle. Exp. Parasitol..

[144] Swenerton R.K., Zhang S., Sajid M., Medzihradszky K.F., Craik C.S., Kelly B.L., McKerrow J.H. (2011). The oligopeptidase B of Leishmania regulates parasite enolase and immune evasion. J. Biol. Chem..

[145] Pinheiro R.O., Pinto E.F., Lopes J.R.C., Guedes H.L.M., Fentanes R.F., Rossi-Bergmann B. (2005). TGF-β-associated enhanced susceptibility to leishmaniasis following intramuscular vaccination of mice with Leishmania amazonensis antigens. Microbes Infect..

[146] Russell D.G., Wilhelm H. (1986). The involvement of the major surface glycoprotein (gp63) of Leishmania promastigotes in attachment to macrophages. J. Immunol..

[147] Bouvier J., Etges R., Bordier C. (1987). Identification of the promastigote surface protease in seven species of Leishmania. Mol. Biochem. Parasitol..

[148] Etges R., Bouvier J., Bordier C. (1986). The major surface protein of Leishmania promastigotes is a protease. J. Biol. Chem..

[149] Pandey S., Chakraborti P., Sharma R., Bandyopadhyay S., Sarkar D., Adhya S. (2004). Involvement of Leishmania donovani major surface glycoprotein gp63 in promastigote multiplication. J. Biosci..

[150] Hey A.S., Theander T.G., Hviid L., Hazrati S.M., Kemp M., Kharazmi A. (1994). The major surface glycoprotein (gp63) from Leishmania major and Leishmania donovani cleaves CD4 molecules on human T cells. J. Immunol..

[151] Colomer-Gould V., Quintao L.G., Keithly J., Nogueira N. (1985). A common major surface antigen on amastigotes and promastigotes of Leishmania species. J. Exp. Med..

[152] Ellis M., Sharma D.K., Hilley J.D., Coombs G.H., Mottram J.C. (2002). Processing and trafficking of Leishmania mexicana GP63. Analysis using GP18 mutants deficient in glycosylphosphatidylinositol protein anchoring. J. Biol. Chem..

[153] Pimenta P.F., Saraiva E.M., Sacks D.L. (1991). The comparative fine structure and surface glycoconjugate expression of three life stages of Leishmania major. Exp. Parasitol..

[154] Brittingham A., Morrison C.J., McMaster W.R., McGwire B.S., Chang K.-P., Mosser D.M. (1995). Role of the Leishmania surface protease gp63 in complement fixation, cell adhesion, and resistance to complement-mediated lysis. J. Immunol..

[155] Brittingham A., Chen G., McGwire B.S., Chang K.-P., Mosser D.M. (1999). Interaction of Leishmania gp63 with cellular receptors for fibronectin. Infect. Immun..

[156] McGwire B.S., Chang K.-P., Engman D.M. (2003). Migration through the extracellular matrix by the parasitic protozoan Leishmania is enhanced by surface metalloprotease gp63. Infect. Immun..

[157] Chaudhuri G., Chaudhuri M., Pan A., Chang K.P. (1989). Surface acid proteinase (gp63) of Leishmania mexicana: A metalloenzyme capable of protecting liposome-encapsulated proteins from phagolysosomal degradation by macrophages. J. Biol. Chem..

[158] Chen D.-Q., Kolli B.K., Yadava N., Lu H.G., Gilman-Sachs A., Peterson D.A., Chang K.-P. (2000). Episomal expression of specific sense and antisense mRNAs in Leishmania amazonensis: Modulation of gp63 level in promastigotes and their infection of macrophages in vitro. Infect. Immun..

[159] Gomez M.A., Contreras I., Halle M., Tremblay M.L., McMaster R.W., Olivier M. (2009). Leishmania GP63 alters host signaling through cleavage-activated protein tyrosine phosphatases. Sci. Signal..

[160] Corradin S., Ransijn A., Corradin G., Roggero M.A., Schmitz A.A., Schneider P., Mauël J., Vergères G. (1999). MARCKS-related protein (MRP) is a substrate for the Leishmania major surface protease leishmanolysin (gp63). J. Biol. Chem..

[161] Blanchette J., Racette N., Faure R., Siminovitch K.A., Olivier M. (1999). Leishmania-induced increases in activation of macrophage SHP-1 tyrosine phosphatase are associated with impaired IFN-γ-triggered JAK2 activation. Eur. J. Immunol..

[162] Jaramillo M., Gomez M.A., Larsson O., Shio M.T., Topisirovic I., Contreras I., Luxenburg R., Rosenfeld A., Colina R., McMaster R.W. (2011). Leishmania repression of host translation through mTOR cleavage is required for parasite survival and infection. Cell Host Microbe.

[163] Bogdan C., Donhauser N., Döring R., Röllinghoff M., Diefenbach A., Rittig M.G. (2000). Fibroblasts as host cells in latent leishmaniosis. J. Exp. Med..

[164] Isnard A., Shio M.T., Olivier M. (2012). Impact of Leishmania metalloprotease GP63 on macrophage signaling. Front. Cell Infect. Microbiol..

[165] Santos A.L., Branquinha M.H., D’Avila-Levy C.M. (2006). The ubiquitous gp63-like metalloprotease from lower trypanosomatids: In the search for a function. Anais Acad. Bras. Ciências.

[166] Chaudhuri G., Chang K.P. (1988). Acid protease activity of a major surface membrane glycoprotein (gp63) from Leishmania mexicana promastigotes. Mol. Biochem. Parasitol..

[167] Moradin N., Descoteaux A. (2012). Leishmania promastigotes: Building a safe niche within macrophages. Front. Cell. Infect. Microbiol..

[168] Xu D., McSorley S.J., Chatfield S.N., Dougan G., Liew F.Y. (1995). Protection against Leishmania major infection in genetically susceptible BALB/c mice by gp63 delivered orally in attenuated Salmonella typhimurium (AroA- AroD-). Immunology.

[169] Reed S.G., Badaro R., Lloyd R.M. (1987). Identification of specific and cross-reactive antigens of Leishmania donovani chagasi by human infection sera. J. Immunol..

[170] Vale A.M., Fujiwara R.T., da Neto A.F.S., Miret J.A., Alvarez D.C., da Silva J.C., Campos-Neto A., Reed S., Mayrink W., Nascimento E. (2009). Identification of highly specific and cross-reactive antigens of Leishmania species by antibodies from Leishmania (Leishmania) chagasi naturally infected dogs. Zoonoses Public Health.

[171] Medina L.S., Souza B.A., Queiroz A., Guimarães L.H., Machado P.R.L., Carvalho E.M., Wilson M.E., Schriefer A. (2016). The gp63 Gene Cluster Is Highly Polymorphic in Natural Leishmania (Viannia) braziliensis Populations, but Functional Sites Are Conserved. PLoS ONE.

[172] Joshi P.B., Sacks D.L., Modi G., McMaster W.R. (1998). Targeted gene deletion of Leishmania major genes encoding developmental stage-specific leishmanolysin (GP63). Mol. Microbiol..

[173] Joshi P.B., Kelly B.L., Kamhawi S., Sacks D.L., McMaster W.R. (2002). Targeted gene deletion in Leishmania major identifies leishmanolysin (GP63) as a virulence factor. Mol. Biochem. Parasitol..

[174] Freitas-Mesquita A.L., Meyer-Fernandes J.R. (2014). Ecto-nucleotidases and Ecto-phosphatases from Leishmania and Trypanosoma parasites. Sub-Cell. Biochem..

[175] Leite P.M., Gomes R.S., Figueiredo A.B., Serafim T.D., Tafuri W.L., de Souza C.C., Moura S.A., Fietto J.L., Melo M.N., Ribeiro-Dias F. (2012). Ecto-nucleotidase activities of promastigotes from Leishmania (Viannia) braziliensis relates to parasite infectivity and disease clinical outcome. PLoS Negl. Trop. Dis..

[176] Joshi M.B., Dwyer D.M. (2007). Molecular and functional analyses of a novel class I secretory nuclease from the human pathogen, Leishmania donovani. J. Biol. Chem..

[177] Boitz J.M., Ullman B. (2006). A conditional mutant deficient in hypoxanthine-guanine phosphoribosyltransferase and xanthine phosphoribosyltransferase validates the purine salvage pathway of Leishmania donovani. J. Biol. Chem..

[178] Peres N.T.A., Cunha L.C.S., Barbosa M.L.A., Santos M.B., de Oliveira F.A., de Jesus A.M.R., de Almeida R.P. (2017). Infection of Human Macrophages by Leishmania infantum Is Influenced by Ecto-Nucleotidases. Front. Immunol.

[179] de Souza M.C., de Assis E.A., Gomes R.S., da Ede A.M.S., Melo M.N., Fietto J.L., Afonso L.C. (2010). The influence of ecto-nucleotidases on Leishmania amazonensis infection and immune response in C57B/6 mice. Acta Trop..

[180] Guimaraes-Costa A.B., DeSouza-Vieira T.S., Paletta-Silva R., Freitas-Mesquita A.L., Meyer-Fernandes J.R., Saraiva E.M. (2014). 3’-nucleotidase/nuclease activity allows Leishmania parasites to escape killing by neutrophil extracellular traps. Infect. Immun..

[181] Vasconcellos Rde S., Mariotini-Moura C., Gomes R.S., Serafim T.D., Firmino Rde C., Silva E.B.M., Castro F.F., Oliveira C.M., Borges-Pereira L., de Souza A.C. (2014). Leishmania infantum ecto-nucleoside triphosphate diphosphohydrolase-2 is an apyrase involved in macrophage infection and expressed in infected dogs. PLoS Negl. Trop. Dis..

[182] Paletta-Silva R., Vieira D.P., Vieira-Bernardo R., Majerowicz D., Gondim K.C., Vannier-Santos M.A., Lopes A.H., Meyer-Fernandes J.R. (2011). Leishmania amazonensis: Characterization of an ecto-3’-nucleotidase activity and its possible role in virulence. Exp. Parasitol..

[183] Feder M.E., Hofmann G.E. (1999). Heat-shock proteins, molecular chaperones, and the stress response: Evolutionary and ecological physiology. Annu Rev. Physiol..

[184] Hombach A., Clos J., Tatu U. (2014). No stress—Hsp90 and signal transduction in Leishmania. Parasitology.

[185] Kröber-Boncardo C., Grünebast J., Clos J. (2020). Heat Shock Proteins in Leishmania Parasites.

[186] Krobitsch S., Clos J. (1999). A novel role for 100 kD heat shock proteins in the parasite Leishmania donovani. Cell Stress Chaperones.

[187] Hübel A., Krobitsch S., Hörauf A., Clos J. (1997). Leishmania major Hsp100 is required chiefly in the mammalian stage of the parasite. Mol. Cell. Biol..

[188] Silverman J.M., Clos J., Horakova E., Wang A.Y., Wiesgigl M., Kelly I., Lynn M.A., McMaster W.R., Foster L.J., Levings M.K. (2010). Leishmania exosomes modulate innate and adaptive immune responses through effects on monocytes and dendritic cells. J. Immunol..

[189] David M., Gabdank I., Ben-David M., Zilka A., Orr I., Barash D., Shapira M. (2010). Preferential translation of Hsp83 in Leishmania requires a thermosensitive polypyrimidine-rich element in the 3’ UTR and involves scanning of the 5’ UTR. RNA.

[190] Young J.C., Agashe V.R., Siegers K., Hartl F.U. (2004). Pathways of chaperone-mediated protein folding in the cytosol. Nat. Rev. Mol. Cell Biol..

[191] Clos J., Krobitsch S. (1999). Heat Shock as a Regular Feature of the Life Cycle of Leishmania Parasites. Am. Zool..

[192] Hombach A., Ommen G., MacDonald A., Clos J. (2014). A small heat shock protein is essential for thermotolerance and intracellular survival of Leishmania donovani. J. Cell Sci..

[193] Wiesgigl M., Clos J. (2001). Heat shock protein 90 homeostasis controls stage differentiation in Leishmania donovani. Mol. Biol. Cell.

[194] Bifeld E., Lorenzen S., Bartsch K., Vasquez J.-J., Siegel T.N., Clos J. (2018). Ribosome profiling reveals HSP90 inhibitor effects on stage-specific protein synthesis in Leishmania donovani. Msystems.

[195] Hombach A., Ommen G., Chrobak M., Clos J. (2013). The Hsp 90–Sti 1 interaction is critical for L eishmania donovani proliferation in both life cycle stages. Cell. Microbiol..

[196] Morales M.A., Watanabe R., Dacher M., Chafey P., Fortéa J.O., Scott D.A., Beverley S.M., Ommen G., Clos J., Hem S. (2010). Phosphoproteome dynamics reveal heat-shock protein complexes specific to the Leishmania donovani infectious stage. Proc. Natl. Acad. Sci. USA.

[197] Adhuna P.S., Salotra P., Mukhopadhyay B., Bhatnagar R. (1997). Modulation of macrophage heat shock proteins (HSPs) expression in response to intracellular infection by virulent and avirulent strains of Leishmania donovani. Biochem. Mol. Biol. Int..

[198] Rico A.I., Del Real G., Soto M., Quijada L., Martinez A.C., Alonso C., Requena J.M. (1998). Characterization of the immunostimulatory properties of Leishmania infantum HSP70 by fusion to the Escherichia coli maltose-binding protein in normal and nu/nu BALB/c mice. Infect. Immun..

[199] Skeiky Y.A., Benson D.R., Guderian J.A., Whittle J.A., Bacelar O., Carvalho E.M., Reed S.G. (1995). Immune responses of leishmaniasis patients to heat shock proteins of Leishmania species and humans. Infect. Immun..

[200] Higgins C.F. (1992). ABC transporters: From microorganisms to man. Annu. Rev. Cell Biol..

[201] Doige C.A., Ames G.F. (1993). ATP-dependent transport systems in bacteria and humans: Relevance to cystic fibrosis and multidrug resistance. Annu. Rev. Microbiol..

[202] Piper R.C., Xu X., Russell D.G., Little B.M., Landfear S.M. (1995). Differential targeting of two glucose transporters from Leishmania enriettii is mediated by an NH2-terminal domain. J. Cell Biol..

[203] Perez-Victoria J.M., Di Pietro A., Barron D., Ravelo A.G., Castanys S., Gamarro F. (2002). Multidrug resistance phenotype mediated by the P-glycoprotein-like transporter in Leishmania: A search for reversal agents. Curr. Drug Targets.

[204] Richard D., Leprohon P., Drummelsmith J., Ouellette M. (2004). Growth phase regulation of the main folate transporter of Leishmania infantum and its role in methotrexate resistance. J. Biol. Chem..

[205] Manzano J.I., Garcia-Hernandez R., Castanys S., Gamarro F. (2013). A new ABC half-transporter in Leishmania major is involved in resistance to antimony. Antimicrob. Agents Chemother..

[206] Prati F., Goldman-Pinkovich A., Lizzi F., Belluti F., Koren R., Zilberstein D., Bolognesi M.L. (2014). Quinone-amino acid conjugates targeting Leishmania amino acid transporters. PLoS ONE.

[207] Parodi-Talice A., Araujo J.M., Torres C., Perez-Victoria J.M., Gamarro F., Castanys S. (2003). The overexpression of a new ABC transporter in Leishmania is related to phospholipid trafficking and reduced infectivity. Biochim. Biophys. Acta.

[208] Ouellette M., Legare D., Haimeur A., Grondin K., Roy G., Brochu C., Papadopoulou B. (1998). ABC transporters in Leishmania and their role in drug resistance. Drug Resist. Updates.

